# Using qualitative exit interviews to explore schizophrenia burden and treatment experience in clinical trial patients

**DOI:** 10.3389/fpsyt.2024.1377174

**Published:** 2024-08-21

**Authors:** Adam Simmons, Amy K. O’Sullivan, Julia Carpenter-Conlin, Michelle K. Carty, Cory Saucier, David McDonnell

**Affiliations:** ^1^ Alkermes, Inc., Waltham, MA, United States; ^2^ QualityMetric, LLC, Johnston, RI, United States; ^3^ Alkermes Pharma Ireland Limited, Dublin, Ireland

**Keywords:** antipsychotic medication, content analysis, drug development, exit interview, patient experience, thematic analysis

## Abstract

**Introduction:**

Qualitative research methods can be used to obtain a deeper understanding of patient experience by collecting information in the patients’ own words about their encounters, perspectives, and feelings. In this study, patients with schizophrenia were interviewed to capture their voice and to complement the quantitative data typically obtained in clinical trials.

**Methods:**

Semi-structured exit interviews were conducted with 41 patients who completed or prematurely discontinued from a phase 3, open-label trial (NCT02873208). The interview guide included open-ended questions on current and prior disease burden, symptoms, quality of life, and treatment experiences. Steps taken to reduce interview stress and secure the validity of data included interviewer sensitivity training specific to mental health conditions and schizophrenia, use of in-person interviews whenever possible and use of videoconferencing for remote interviews to promote trust and comfort, and working closely with clinical site staff to identify patient eligibility and willingness to participate. Transcripts based on audio recordings were content coded and analyzed using thematic analysis; a *post-hoc* quantitative content analysis was conducted.

**Results:**

Patients reported that the symptoms of schizophrenia negatively impacted their work, relationships, self-esteem, emotional health, and daily activities. Most patients had positive experiences with medications that alleviated hallucinations, depression, and anxiety. However, side effects of medications were associated with negative impacts on physical, emotional, behavioral, and cognitive health. Lack of energy/drowsiness, weight gain, mood changes, and involuntary movements were the most common side effects reported with the use of antipsychotic medications. Patients reported unmet treatment needs related to better symptom control and to improved social and physical functioning.

**Conclusion:**

Collection of qualitative information within a schizophrenia clinical development process provides value and insights into patients’ views on burden of illness, experiences with previous medications, and experiences following participation in a clinical trial and can inform design for future studies.

## 1 Introduction

Schizophrenia is a potentially debilitating mental disorder that is associated with substantial disease burden, such as the presence of physical and mental health comorbidities and the risk of premature mortality (1–4). Living with schizophrenia is also associated with increased healthcare resource use and high medical costs compared with those living in the general population (5, 6). In addition, the symptoms of schizophrenia may have negative consequences on obtaining gainful employment, quality of life, and social relationships (7, 8).

In schizophrenia research, treatment outcomes are generally assessed by clinician ratings and/or symptom scales. These quantitative evaluations play an important role in the overall review of drug effectiveness (9). For example, research has indicated that long-term antipsychotic treatment of schizophrenia is associated with decreased disease burden and improvements in quality of life and social functioning based on assessments with published rating scales (10, 11). As the information gleaned from rating scales is generally focused on patient symptoms and established measures of quality of life, it can be somewhat limited in scope.

The US Food and Drug Administration has encouraged the use of qualitative research methods to ascertain patient perspectives in the process of drug development (12). Novel methodology can be used to obtain a deeper understanding of the patient experience by exploring perspectives and feelings using the patient’s own words (12). The adaptability of the qualitative research design facilitates patients’ ability to reliably share their experiences. With respect to schizophrenia, qualitative methodologies can help researchers capture nuances related to disease burden, daily functioning, and treatment experiences that otherwise may not be collected in quantitative assessments during clinical trials.

Herein, we describe a qualitative exit interview that was conducted in patients living with schizophrenia who had participated in a 52-week, phase 3, open-label extension study. We sought to better understand the burden of illness associated with schizophrenia in patients’ own words, as well as their experiences with previous and current treatments and how they have impacted their daily life.

## 2 Methods

### 2.1 Study design

**Figure 1:**
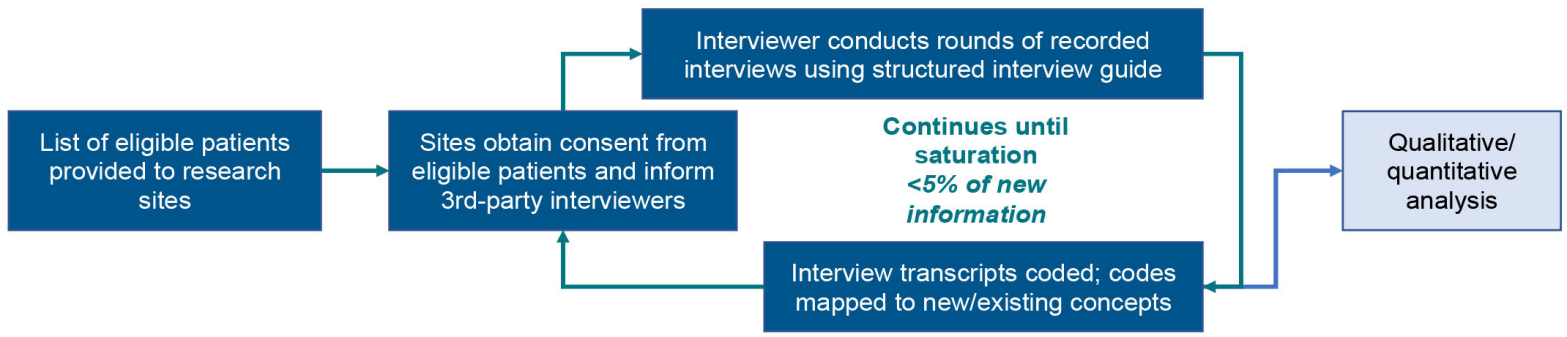
Study design.

This was a qualitative, non-interventional interview of patients with schizophrenia who completed or prematurely discontinued from the open-label ENLIGHTEN-2 extension study (ENLIGHTEN-2-EXT; ClinicalTrials.gov identifier: NCT02873208), and was included as an appendix to complement the main study protocol, which was approved by the Copernicus Group Institutional Review Board (CGIRB). This exploratory qualitative method was selected in order to explore spontaneous patient descriptions of the disease burden and treatment experiences of patients living with schizophrenia prior to and during participation in the study. Special attention was focused on the interview process (described below) to ensure that the patient’s voice was captured constructively. The qualitative interview design is illustrated in **Figure 1**.

### 2.2 Study participants

Patients were adults aged 18 or older with a primary diagnosis of schizophrenia who either completed or terminated study participation early and who were willing and able to provide written informed consent for the interview. Further details of inclusion/exclusion criteria are available within the primary study results reported elsewhere (13, 14). Additionally, patients were required to speak English fluently, be willing and able to participate in a 60-minute interview, either in person or via telephone, and agreed to schedule the interview within 60 days of completing or discontinuing from the study.

### 2.3 Interview procedures

Interviews were conducted by trained qualitative researchers who had experience working with individuals with mental illness. All interviews lasted approximately 60 minutes. Interviews could take place either in person at the study sites, or via telephone or videoconference. Patient interviews were conducted within 60 days of completing or discontinuing from the study. All interviews were audio-recorded and transcribed.

Interviewers followed a semi-structured interview guide that was developed specifically for this interview. A draft of the interview guide content was informed by findings from a targeted literature review and from a subsequent review by 3 clinicians experienced in the management of patients with schizophrenia and familiarity with study procedures and the interview. The interview guide included open-ended questions designed to allow patients to spontaneously report their experiences related to disease burden and symptoms, quality of life, prior treatments, and study-related treatment experiences, both current and prior. Before conducting the first interviews, the interview guide was submitted to and approved by the CGIRB. After the first round of interviews (n=7) was completed, transcribed, and analyzed according to procedures, minor changes were made to the guide to better capture emergent concepts and pose questions. The amended guide was then approved by the CGIRB, and no additional changes were made after subsequent interview rounds.

**Figure 2:**
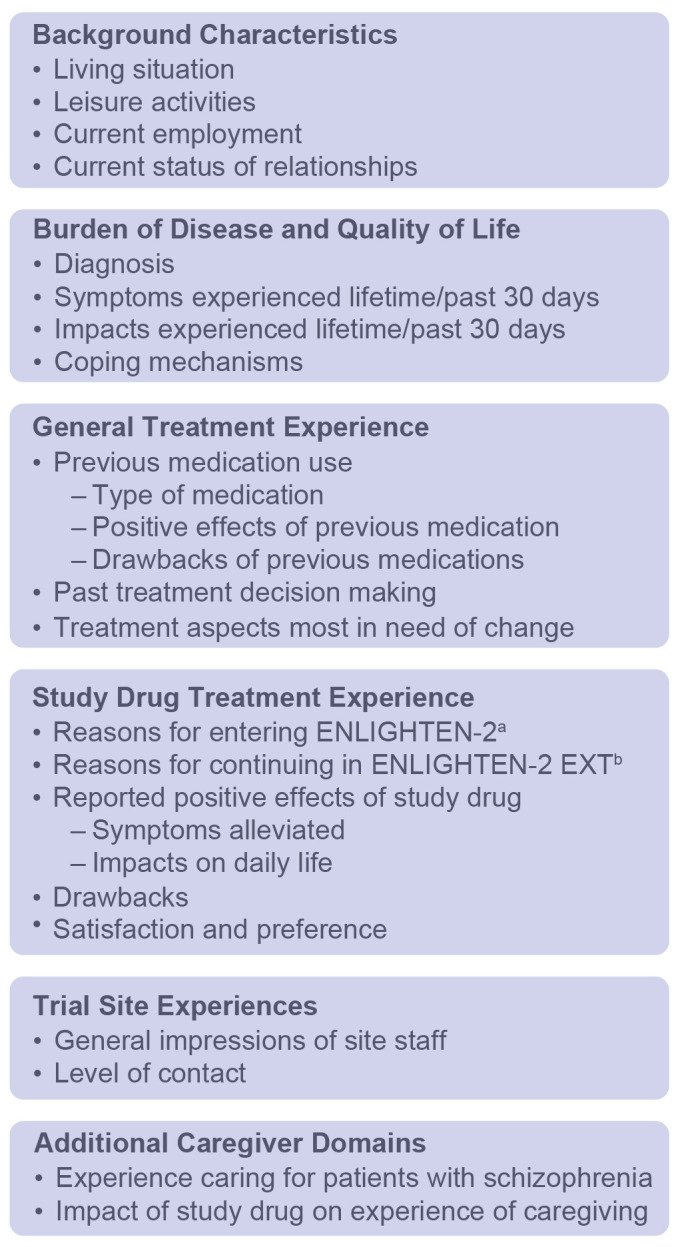
Interview Guide Domains. ^a^24-week, phase 3, double-blind study. ^b^52-week, phase 3, open-label extension of ENLIGHTEN-2.

The interview guide included questions on the following domains regarding burden of disease: (1) initial diagnosis, (2) average day, (3) symptoms, including the past 30 days, (4) impact of symptoms on daily life, and (5) coping strategies (**Figure 2**). Questions within the following domains assessed treatment experience: (1) prior treatment experiences and preferences, (2) study-specific treatment experiences and preferences, (3) impact of treatment on quality-of-life domains, and (4) comparative satisfaction with current study treatment versus prior treatment experiences (**Figure 2**).

Researchers employed several strategies to support data collection, including (1) using highly experienced interviewers and providing interviewer sensitivity training specific to mental health conditions and schizophrenia, (2) initially conducting a limited number of interviews at the first clinical sites to identify any needed modifications to the protocol or interview guide, (3) conducting interviews in-person whenever possible, (4) holding interviews where clinically trained staff are available for patient safety, (5) using a list of common antipsychotic medications to aid recall, (6) making changes to the interview guide questions to ensure sensitivity and clarity, (7) using interviewer redirection to minimize excessive digression during conversation, (8) allowing for the use of videoconferencing for interviews conducted remotely, to promote trust and comfort for the patient, and (9) working closely with clinical site staff to identify patient eligibility for scheduling.

### 2.4 Coding and analysis

All interviews were audiotaped, transcribed, coded, and subsequently analyzed. Transcripts based on audio recordings were content coded and analyzed using thematic analysis, and a *post-hoc* quantitative content analysis was conducted (15, 16). There were 2 parts to the analysis: qualitative data coding and qualitative data analysis. The process of coding is necessary to construct themes that emerge. All qualitative analyses were conducted using NVivo, version 11.0 (Burlington, MA, USA). Interviews were analyzed and compared in 6 rounds of coding until no new concepts emerged from collected data and saturation was achieved. Analysis of the interviews occurred in 2 iterative phases: (1) development of coding categories and (2) organization of codes into larger themes, as described below.

Following the first set of patient interviews (n=7), 2 researchers independently coded, reviewed, and analyzed the transcripts to identify relevant concepts associated with the interview objectives, developing an initial set of codes for each transcript. Thereafter, consensus meetings between the 2 coders and the principal investigator were held to develop a codebook that included operational definitions and guidelines for coding subsequent transcripts. Discrepancies between coders were discussed until a consensus was reached.

In subsequent interview rounds, 1 researcher coded each set of transcripts and a second researcher reviewed the coding. Consensus meetings were held throughout this process to ensure agreement among the research team. If necessary, the codebook was revised to address any newly emergent codes. When this occurred, re-analysis of prior rounds of interviews was conducted to determine whether any content in those rounds could be identified that was covered by the newly emergent codes.

### 2.5 Saturation analysis

A formal saturation analysis of patient data was conducted with a preset criterion that saturation would be reached when new codes generated in the final round of interviews constituted fewer than 5% of all codes. As new codes were discovered and/or confirmed by subsequent data collection, researchers grouped and organized them according to larger themes and patterns. Codes and themes were further analyzed, refined, and combined into a distinct theoretical framework that described the relationships among identified themes. It was estimated that approximately 50 patients would be required to reach saturation.

## 3 Results

A total of 41 patients were interviewed, at which point saturation of new codes was achieved. Patient demographics and background characteristics are detailed in **Table 1** and **Supplementary Table S1**, respectively. The mean age of patients interviewed was 44.2 years old; the majority of those interviewed were male and black, which was representative of the study population (14). Most patients lived with relatives and were unemployed. With the exception of one patient, all patients (n=40) completed the extension trial. Twenty-four (58.5%) interviews took place in person, with the remainder conducted via videoconference or telephone. Overall, findings from the saturation analysis confirmed that 41 interviews were sufficient to reach saturation, as fewer than 5% of all concepts emerged during the last round of interviews (ie, interviews 36 to 41) (**Table 2**).

**Table 1:** Patient demographics.

Demographic	Patients (N=41)
Age, mean (SD)	44.2 (9.5)
Sex, n (%)	
Male	25 (61.0)
Female	16 (39.0)
Race, n (%)	
Black	33 (80.5)
White	8 (19.5)
Ethnicity, n (%)	
Not Hispanic or Latino	37 (90.2)
Hispanic or Latino	4 (9.8)
Interview type, n (%)	
In-person	24 (58.5)
Videoconference or telephone call	17 (41.5)
Patient study status, n (%)	
A304 completer/A308 rollover	40 (97.6)
A304 early terminated	1 (2.4)

**Table 2:** Saturation analysis.

Major Theme, no.^a^	Code Set 1 (int 1-7)	Code Set 2 (int 8-14)	Code Set 3 (int 15-21)	Code Set 4 (int 22-28)	Code Set 5 (int 29-35)	Code Set 6 (int 36-41)
Diagnosis	3	1	–	–	–	–
Symptoms experienced, lifetime	21	10	1	–	–	2
Impacts						
Daily activities	3	3	1	–	–	–
Emotional health	13	10	8	–	1	2
Ability to work	3	1	1	–	2	1
Productivity at work	4	2	–	–	1	1
Physical functioning	11	7	3	–	–	2
Relationships	8	2	3	–	–	–
Social	7	7	1	1	2	–
Other	6	1	1	–	–	1
Coping mechanisms^b^						
Positive	23	7	6	4	2	6
Negative	4	1	3	–	–	–
Medication use	18	8	2	6	1	–
Changes in schizophrenia symptoms	21	5	3	1	3	1
Changes in impacts						
Daily activities	7	2	–	1	2	–
Alleviated side effects	2	6	2	2	3	–
Emotional health	8	2	2	3	1	2
Leisure activities	4	2	–	–	–	–
Physical functioning	5	2	1	–	–	1
Relationships	3	–	–	1	–	–
Social	9	1	1	2	–	–
Work	1	–	–	–	–	–
Other	10	3	4	2	–	–
Study drug treatment experiences^c^	17	5	3	5	4	2
ALKS3831-A303/A304 trials						
Reasons for entering	6	6	4	2	1	—
Reasons for continuing	13	2	8	3	3	—
Treatment preferences	1	1	1	—	1	1
Total new codes (% of new codes per set)	231 (49.3)	97 (20.7)	59 (12.6)	33 (7.0)	27 (5.8)	22 (4.7)

Int, interview.

a Number of new codes identified within each set of interviews.

b Positive and negative coping mechanisms were categorized based on patient reports.

c Treatment experiences include patient reports of positive effects, drawbacks and side effects, and level of satisfaction with study drug treatment.

### 3.1 Schizophrenia symptoms and their impact

Among the 41 patients interviewed, auditory hallucinations were the most common symptom experienced at the time of diagnosis, being reported by 73.2% of patients (**Supplementary Table S2**). In addition, paranoia, visual hallucinations, and thoughts of self-harm were endorsed by approximately 30% of interviewees at the time of the initial diagnosis. Referring to initial experiences with auditory hallucinations, one patient stated:

“My initial issues were just … I mean, I had the voices … I was worried … break into my apartment, try to hurt me or something. Wondering where the voices were coming from … I was thinking the voices were coming [from] outside my head.”

The most common impact of initial symptoms was hospitalization, with nearly 44% of patients reporting this outcome. Additionally, schizophrenia symptoms at the time of diagnosis led to substance use in 14.6% and a nervous breakdown/psychotic episode in 12.2% of those interviewed.

Consistent with experiences at the initial diagnosis, auditory hallucinations, visual hallucinations, and paranoia were also the most frequently reported schizophrenia symptoms experienced during the last 30 days and over the patient’s lifetime (**Supplementary Table S3**). A patient discussed the experience of paranoia by saying:

“I’d be cautious around everything, even the people I’m close to. It’s like I don’t know what’s about to happen and I start shaking and start sweating and get really hot and I just … and then I just start questioning everything.”

Although anxiety was reported at initial diagnosis in only 7.3% of patients interviewed (**Supplementary Table S2**), anxiety and worry became more common over time and were reported by 22% of patients within the past 30 days and by 31.7% over the patient’s lifetime (**Supplementary Table S3**).

All patients described the burden of schizophrenia symptoms on their quality of life. **Supplementary Table S4** details the negative impact of symptoms of schizophrenia over the last 30 days and lifetime on various aspects of the patient experience. Most frequently, these impacts were related to work (82.9%), hospitalizations [eg, number of admissions to a hospital (73.2%)], relationships (70.7%), self-esteem (65.9%), social isolation (65.9%), emotional health (61.0%), and daily activities (51.2%). With respect to emotional health, one patient said:

“Depression. It puts me down because I don’t act normal. Like I said, all my friends are married, went off to college, played football, became schoolteachers, and I feel like I’m the underachiever. I never really felt like I really fit in as normal because I was taking medications and stuff. Especially early in my 20s, when most kids are finishing college and making their choices on what they want to be in life, and that’s kind of embarrassing to me. It’s kind of embarrassing.”

### 3.2 Coping mechanisms

All patients described behaviors and strategies for coping with symptoms of schizophrenia. Both positive and negative coping mechanisms were reported by patients (**Supplementary Table S5**). The most common positive coping mechanisms were entertainment (53.7%), social activities/social support (34.1%), physical activity (31.7%), and mindfulness/meditation (29.3%). A patient described how playing video games provided a positive way to cope with his symptoms:

“I’m a huge video gamer. I play a lot. I have like 45 games for my PS4 and I have 12 games to my Nintendo Switch. I play video games because they keep me focused and the competition, competitiveness in the game keeps me focused on that, including reading books, watching DVDs and things like that.”

The most common negative coping mechanisms were smoking cigarettes (39.0%), alcohol/marijuana use (12.2%), and self-isolation (9.8%).

One patient described the calming effect of smoking as follows:

“I smoke cigarettes. A lot of people don’t realize when you’re real mad and you smoke cigarettes your attitude changes, it goes for the better. You smoke a cigarette, you’ll calm down.”

### 3.3 Past treatment experiences

The majority of patients (78.0%) reported positive experiences with previous medications taken, with alleviation of auditory/visual hallucinations, depression, and anxiety reported in 68.8%, improvements in sleep in 34.4%, and feeling calmer in 25.0% of patients interviewed. Patients reported 35 distinct side effects related to past medication use (**Supplementary Table S6**). Overall, 95.1% experienced side effects from past medications that impacted their physical health, 41.5% experienced emotional or behavioral impacts, and 12.2% reported impacts on cognitive health. Lack of energy/drowsiness and weight gain were the most common impacts to physical health. When discussing lack of energy/drowsiness, one patient explained:

“Yeah, all day, you never feel completely awake. With that, I stay asleep a lot. I didn’t function, I wasn’t able to function like I do now. That was bad, because you go, you’re sleeping or you’re tired, or you could tell you’re tired, or—you just drag all day.”

Emotional and behavioral impacts of past treatments included changes in mood and increased appetite or compulsive eating, respectively. Cognitive impacts included not thinking clearly, slurred or stuttering speech, and being forgetful. Ineffectiveness of the medication was reported by 31.7% of those interviewed.

### 3.4 Remaining treatment needs

When patients with schizophrenia were asked about aspects of schizophrenia that they would like medications to improve, the most common response was alleviation of hallucinations, which was endorsed by 60.5% of respondents. Additionally, patients with schizophrenia wanted medications to improve social functioning and the ability to maintain relationships, improve physical function, and reduce paranoia. Primary and secondary treatment aspects reported as the most in need of change are listed in **Supplementary Table S7**.

### 3.5 Treatment experience

#### 3.5.1 Reasons for entering study

Patients included in this interview reported entering the original study for several reasons, including the desire to find a medication that provided better symptom control, following a recommendation from others to participate, the financial incentive to participate, and the wish to be of benefit to others. One patient stated that achieving better symptom control was the reason for their participation:

“To see if the medication can help or I can get some better results from it.”

Another patient discussed in detail how a friend convinced them to participate in the study:

“I chose to go into it because I had a friend who, she was participating in the research, and she said it’s pretty helpful and good to go to the study thing. They might help you adjust your medication, what you get right now, and try this and stuff. I said, ‘Okay.’ And then I felt like it was helping, so that’s why I started coming here. My friend recommended me to come in.”

The top reasons for continuing into the extension study, where patients received open-label treatment, included the effectiveness of alleviating symptoms (53.7%) and experiencing fewer side effects compared with previous medications (17.1%).

#### 3.5.2 Symptom management

Patient interviews reflected an improvement in 16 total symptoms as coded by reviewers since the initiation of treatment in the study. The most frequently coded improvements were alleviation of, or a reduction in frequency or intensity of, auditory hallucinations (63.4%; **Supplementary Table S8**). When describing how the study treatment helped their auditory hallucinations, one patient said:

“It helps me to alleviate the intensity of the voices … When I say ‘intensity,’ I am speaking of the volume, the strength of it when they are talking these derogatory conversations and comments about me. Yeah. I did not hear that high pitch. There is a woman and she has a really vulgar voice. I mean it is really wrenching in a high pitch and that really helped to lessen it, if that makes sense.”

Additionally, patients suggested that treatment was associated with improvements in concentration (34.1%), along with reductions in paranoid thinking (31.7%), anxiety (22.0%), depression (19.5%), and visual hallucinations (17.1%) (**Supplementary Table S8**). The benefit of the study treatment on concentration was explained by one patient as follows:

“I just get up and I do not feel scattered. I can focus and accomplish what I need. Before that, I was just kind of like a leaf in a big wind. I just went where the breeze took me. It is getting back to some sense of normality now because of the med so it is cool.”

#### 3.5.3 Drawbacks

Overall, 56.1% of patients reported drawbacks with study treatment, while 43.9% reported that there were none. The most common drawbacks were a lack of energy (29.3%), followed by dry mouth (12.2%). A patient explained their experience with these drawbacks by saying:

“The medicine I’m on now it kind of gives me dry mouth in the morning. It kind of gets me a little bit groggy, but I tend to go in the kitchen, make me something to eat, get me a drink of orange juice and I’m good to go then.”

### 3.6 Impact on daily life

Patient interviews indicated an improvement in a total of 16 different aspects of patients’ lives since initiating study treatment. Most patient statements reflected a change in emotional or mental well-being (63.4%), self-esteem (61.0%), social activity (61.0%), relationships (51.2%), daily activities (46.3%), and sleep (41.5%; **Supplementary Table S9**). With respect to the positive effect of study treatment on self-esteem, a patient said:

“Yeah, it did help my self-esteem greatly and started getting me out of the house as opposed to not taking the medication or taking other medications in the past where they would leave me uncomfortable, you know?”

In the opinion of another patient, the study treatment made it easier to engage in daily activities that were previously challenging:

“I can get up and clean my living room, scrub my toilets and stuff, scrub the bathtub and stuff. You know before I wouldn’t do none of that stuff … take out the trash, go shopping, cook. I couldn’t do all that stuff before because I didn’t feel like doing it.”

One patient noted improvements in overall well-being since initiating the study treatment:

“It makes me feel good. I participate more. I smile more.”

### 3.7 Satisfaction and preference

Overall, 87.5% of patients provided survey answers indicating they were “satisfied” or “very satisfied” with treatment; this proportion was higher than satisfaction reported with prior medications. In total, 85.4% of respondents indicated a desire to continue study treatment if possible. One patient noted that they would continue receiving treatment to remain stable:

“I would continue. I would continue. It’s been doing so good, I wouldn’t want to change it right now.”

Another patient discussed their satisfaction with the study treatment in terms of the absence of side effects and feeling like their self:

“It helps. It really helps me. Keeps me at a peace. It don’t give me no bad side effects. It allows me to be able to go to sleep at night regularly, normal. Without tossing and turning. It allows me to wake up feeling like myself.”

## 4 Discussion

Individual patient exit interviews were conducted to gain a better understanding of the life experience of patients with schizophrenia, including their burden of living with schizophrenia, experiences with past treatments, and perspectives on study treatment. Auditory hallucinations, visual hallucinations, and paranoia were common symptoms that patients experienced during the course of their illness. Patients reported that the symptoms of schizophrenia interfered with work, led to hospitalizations, strained social relationships, and negatively affected their emotional health. Previous medications were beneficial in alleviating the symptoms of schizophrenia, but side effects, such as lack of energy and weight gain, were common.

Overall, patients wanted antipsychotic medications that could alleviate their hallucinations and improve social and physical functioning. When asked why they participated in the original open-label extension study, patients reported that it was because of the effectiveness of the study treatment at alleviating symptoms, as well as the experience of fewer side effects compared with previous medications. Patients noted that the study treatment reduced the intensity of their auditory hallucinations and managed other symptoms of schizophrenia, including paranoia, anxiety, and depression.

Patients also reported improvements in several aspects of daily life since initiating study treatment, such as emotional and mental well-being, self-esteem, social activities, relationships, and daily functioning.

Almost all interviewed patients had experienced physical side effects with previous medication, and many also reported side effects that affected their emotional/behavioral or cognitive health. Drawbacks of current treatment included side effects such as lack of energy and dry mouth. However, more than 40% of patients experienced no side effects with the study medication. Most patients reported that they were satisfied or very satisfied with the study treatment and wanted to continue it if possible.

The use of novel, qualitative research methods as outlined in this paper illustrates the feasibility of providing additional insights into the patient experience that supplements findings from data collected in the parent clinical trial. For example, the parent clinical trial evaluated schizophrenia symptoms over time using the Positive and Negative Syndrome Scale (PANSS; a validated, quantitative, and clinically relevant outcome measure commonly used in schizophrenia research); total scores were relatively stable over the duration of the study. However, in the qualitative research, which was conducted at a separate time as were PANSS assessments, patients reported improvements in several aspects of their daily lives, including self-esteem, social activities, and relationships. Therefore, the interview process may identify additional benefits of a medication beyond those captured by traditional clinical measures and may highlight challenges that patients with schizophrenia experience during study treatment. This, in turn, may help clinicians gain a deeper understanding of a medication’s impact on a patient’s daily life and well-being. For patients, participation in such interviews, where their thoughts and feelings regarding schizophrenia and its treatment are explored more extensively than might otherwise occur in a typical office visit, may facilitate better communications with their own healthcare providers and empower them to actively participate in treatment decisions regarding their own care.

When interviewing patients who may have difficulties with verbal and emotional expression (17), facilitating interactions that are more conversational in nature is an important consideration. The semi-structured interview guide provided flexibility to adapt the interview based on observed patient responses and characteristics. Additionally, by not positing a hypothesis *a priori*, this design allowed for new concepts related to study treatment to spontaneously emerge; the methodology ensured that concepts of importance and relevance to the patient were elicited, a feature unique to this interview design and not possible with many patient-reported outcome surveys or other quality of life instruments. As is typical with qualitative studies, modification of the interview guide after the first round of interviews allowed for incorporation of learnings from earlier interviews to maximize efficient data collection.

Several limitations should be considered. First, this was an open-label treatment study; therefore, positive treatment responses may be more likely due to both study participation and treatment with an active agent. Ideally, patient interviews should be included in double-blind studies to also test for and compare differences in responses between treatment groups. Secondly, because 40 of the 41 patients interviewed completed the study, the sample may be biased toward patients who had positive experiences and, therefore, remained on treatment rather than discontinuing from the study. Additionally, as the study enrolled patients with stable symptoms of schizophrenia appropriate for outpatient management, the results observed here may not generalize to patients in whom schizophrenia symptoms are more severe. Furthermore, although the interview format allowed for flexibility in terms of interview questioning, it may have led to missing data, as not every patient was asked the same questions. Lastly, recall challenges may have occurred given the design, and no causal inferences can be made. However, recall bias was mitigated by adding a list of oral antipsychotic medications to the interview guide after the first round of interviews in case any patient had struggled naming previous medications taken. As a result, only one patient was unable to name or describe previous medication. Including a baseline assessment in the design also may have helped mitigate recall bias.

## 5 Summary

The innovative approach taken in this interview provided detailed and valuable insights into the burden of illness in schizophrenia, patients’ experiences with previous medications, and experiences following participation in a clinical trial. Results of this qualitative analysis offer supportive data on patient experiences from a subgroup of patients participating in a long-term, open-label extension study. These findings provide important additional information that complement the quantitative efficacy and safety measurements obtained in the ENLIGHTEN-2 extension study. Insights into the potential benefits and challenges of this study treatment, as identified by the patients themselves, can also inform our selection of study endpoints in future clinical trials in this patient population.

## Data Availability

The datasets presented in this article are not readily available because the data collected in this study are proprietary to Alkermes, Inc. Alkermes, Inc. is committed to public sharing of data in accordance with applicable regulations and laws, and requests can be submitted to the corresponding author. Requests to access the datasets should be directed to David.McDonnell@alkermes.com.

## References

[1] Correll CU Solmi M Croatto G Schneider LK Rohani-Montez SC Fairley L. Mortality in people with schizophrenia: a systematic review and meta-analysis of relative risk and aggravating or attenuating factors. World Psychiatry. (2022) 21:248–71. doi: 10.1002/wps.20994 PMC9077617 35524619

[2] Mitchell AJ Vancampfort D Sweers K van Winkel R Yu W De Hert M. Prevalence of metabolic syndrome and metabolic abnormalities in schizophrenia and related disorders—a systematic review and meta-analysis. Schizophr Bull. (2013) 39:306–18. doi: 10.1093/schbul/sbr148 PMC3576174 22207632

[3] Buckley PF Miller BJ Lehrer DS Castle DJ. Psychiatric comorbidities and schizophrenia. Schizophr Bull. (2009) 35:383–402. doi: 10.1093/schbul/sbn135 19011234 PMC2659306

[4] Crump C Winkleby MA Sundquist K Sundquist J. Comorbidities and mortality in persons with schizophrenia: a Swedish national cohort study. Am J Psychiatry. (2013) 170:324–33. doi: 10.1176/appi.ajp.2012.12050599 23318474

[5] Cloutier M Aigbogun MS Guerin A Nitulescu R Ramanakumar AV Kamat SA. The economic burden of schizophrenia in the United States in 2013. J Clin Psychiatry. (2016) 77:764–71. doi: 10.4088/JCP.15m10278 27135986

[6] Lafeuille MH Dean J Fastenau J Panish J Olson W Markowitz M. Burden of schizophrenia on selected comorbidity costs. Expert Rev Pharmacoecon Outcomes Res. (2014) 14:259–67. doi: 10.1586/14737167.2014.894463 24593801

[7] Crespo-Facorro B Such P Nylander AG Madera J Resemann HK Worthington E. The burden of disease in early schizophrenia—a systematic literature review. Curr Med Res Opin. (2021) 37:109–21. doi: 10.1080/03007995.2020.1841618 33095689

[8] Doane MJ Raymond K Saucier C Bessonova L O’Sullivan AK White MK. Unmet needs with antipsychotic treatment in schizophrenia and bipolar I disorder: patient perspectives from qualitative focus groups. BMC Psychiatry. (2023) 23:245. doi: 10.1186/s12888-023-04746-4 37046256 PMC10091535

[9] Mortimer AM. Symptom rating scales and outcome in schizophrenia. Br J Psychiatry Suppl. (2007) 50:s7–14. doi: 10.1192/bjp.191.50.s7 18019038

[10] American Psychiatric Association. The american psychiatric association practice guideline for the treatment of patients with schizophrenia. Washington, DC: American Psychiatric Association (2019).

[11] Leucht S Leucht C Huhn M Chaimani A Mavridis D Helfer B. Sixty years of placebo-controlled antipsychotic drug trials in acute schizophrenia: systematic review, bayesian meta-analysis, and meta-regression of efficacy predictors. Am J Psychiatry. (2017) 174:927–42. doi: 10.1176/appi.ajp.2017.16121358 28541090

[12] Principles for selecting, developing, modifying, and adapting patient-reported outcome instruments for use in medical device evaluation; guidance for industry and Food and Drug Administration staff, and other stakeholders; availability. FDA-2020-D-1564. Fed Regist. (2022) 87:4033–5. Available at: https://www.fda.gov/media/141565/download.

[13] Correll CU Newcomer JW Silverman B DiPetrillo L Graham C Jiang Y. Effects of olanzapine combined with samidorphan on weight gain in schizophrenia: a 24-week phase 3 study. Am J Psychiatry. (2020) 177:1168–78. doi: 10.1176/appi.ajp.2020.19121279 32791894

[14] Kahn RS Silverman BL DiPetrillo L Graham C Jiang Y Yin J. A phase 3, multicenter study to assess the 1-year safety and tolerability of a combination of olanzapine and samidorphan in patients with schizophrenia: results from the ENLIGHTEN-2 long-term extension. Schizophr Res. (2021) 232:45–53. doi: 10.1016/j.schres.2021.04.009 34015555

[15] Braun V Clarke V. Using thematic analysis in psychology. Qual Res Psychol. (2006) 3:77–101. doi: 10.1191/1478088706qp063oa

[16] Holsti OR. Content analysis. In: Lindzey G Aronson E, editors. Handbook of Social Psychology., 2nd ed. Addison-Wesley, Reading, MA (1968). p. 596–692.

[17] Correll CU Schooler NR. Negative symptoms in schizophrenia: a review and clinical guide for recognition, assessment, and treatment. Neuropsychiatr Dis Treat. (2020) 16:519–34. doi: 10.2147/ndt.S225643 PMC7041437 32110026

