# Supplementary material for: Using qualitative exit interviews to explore schizophrenia burden and treatment experience in clinical trial patients
Source: Front Psychiatry. 2024 Aug 21;15:1377174. doi: 10.3389/fpsyt.2024.1377174 (PMC11372304; doi:10.3389/fpsyt.2024.1377174)
Supplement: Supplementary file 1 [file Datasheet1.pdf]

Supplementary Material

SUPPLEMENTAL TABLES

Table S1. Background Characteristics

Parameter	Participants (N=41)
**Living Situation, n (%)**	
Lives with relative(s)	13 (31.7)
Parent	7 (17.1)
Sibling	3 (7.3)
Other	3 (7.3)
Lives with significant other and/or children	10 (24.4)
Lives alone	10 (24.4)
Lives with roommates	4 (9.8)
Lives in transitional housing	4 (9.8)
**Current Employment Status, n (%)**	
Does not work	30 (73.2)
Unspecified income source	17 (41.5)
Disability/social security[a]	10 (24.4)
Currently in school	2 (4.9)
Works	11 (26.8)
Landscaping	2 (4.9)
Unspecified	2 (4.9)
Boarding home	1 (2.4)
Electrician work	1 (2.4)
Gas station	1 (2.4)
Mental health field	1 (2.4)
Restaurant	1 (2.4)
Security	1 (2.4)
Warehouse	1 (2.4)
**Hobbies/leisure activities,[b,c] n (%)**	
Watches television (including sports and movies at home)	22 (53.7)
Physical activities (eg, playing sports, hiking, walks)	14 (34.1)
Reading/writing	9 (22.0)
Playing games (video games & board games)	8 (19.5)
Going to the movie theater	7 (17.1)
Shopping	6 (14.6)
Music (singing, recording, listening)	5 (12.2)
Attending church	4 (9.8)
Cooking at home	4 (9.8)
Going out to eat	4 (9.8)
Painting	2 (4.9)
Remodeling home	2 (4.9)
No hobbies reported	2 (4.9)

[a]Five patients reported being on “disability” and 5 reported being on “social security”.
[b]Hobbies participated in by only 1 patient: working on car, riding bus, traveling, theme parks/zoo, visiting friends, sitting outside in wheelchair, riding motorcycle, picnics, and sitting by the water.
[c]Patients may have reported more than 1 activity.

Table S2. Initial Symptoms at Time of Schizophrenia Diagnosis

Symptoms, n (%)	Participants (N=41)[a]
Auditory hallucinations	30 (73.2)
Paranoia	13 (31.7)
Visual hallucinations	12 (29.3)
Harmful thoughts/behaviors	12 (29.3)[b]
Suicidal thoughts/self-harm	7 (17.1)
Violence toward others	5 (12.2)
Constant anger	1 (2.4)
Depression	4 (9.8)
Anxiety/agoraphobia	3 (7.3)
Delusions	2 (4.9)
Mood swings	1 (2.4)
Racing thoughts	1 (2.4)
Negative self-talk	1 (2.4)

[a]Patients may have reported more than one symptom.

[b]One patient reported suicidal ideation and violence toward others.

Table S3. Symptoms Experienced: Lifetime and Past 30 Days

	Lifetime	Last 30 Days
**Symptoms, n (%)**	N=41[a]	N=41[a]
Auditory hallucinations	36 (87.8)	18 (43.9)
Paranoia	24 (58.5)	9 (22.0)
Visual hallucinations	18 (43.9)	8 (19.5)
Anxiety/worry/panic	13 (31.7)	9 (22.0)
Mad easily/mood	11 (26.8)	3 (7.3)
Delusions	6 (14.3)	2 (4.9)
Negative thoughts/urges	5 (12.2)	3 (7.3)
Olfactory hallucinations	1 (2.4)	0 (0)
Flat affect	1 (2.4)	0 (0)
No symptoms reported	0 (0)	9 (22.0)

[a]Patients may have reported more than one symptom.

Table S4. Schizophrenia Symptom Impacts: Lifetime and Last 30 Days

Impacts,[a] n (%)	Lifetime (N=41)[b]	Last 30 Days (N=41)
Work	34 (82.9)	—
Hospitalization	30 (73.2)	0 (0)
Relationships	29 (70.7)	—
Self-esteem	27 (65.9)	0 (0)
Social isolation/avoiding others	27 (65.9)	9 (22.0)
Emotional health	25 (61.0)	2 (4.9)
Daily activities	21 (51.2)	1 (2.4)
Physical health	13 (31.7)	3 (7.3)
Cognitive difficulties/concentration	13 (31.7)	5 (12.2)
Suicidality	13 (31.7)	0 (0)
Trouble sleeping/bad dreams	13 (31.7)	4 (9.8)
Substance use	9 (22.0)	4 (9.8)
Trouble communicating	6 (14.6)	0 (0)
Violent thoughts/behaviors	6 (14.6)	0 (0)
Ability to do leisure activities	5 (12.2)	3 (7.3)
Education	5 (12.2)	0 (0)
Panic attacks/nervous breakdown	4 (9.8)	1 (2.4)
Homelessness	3 (7.3)	0 (0)
Incarceration	3 (7.3)	0 (0)
Motivation	3 (7.3)	0 (0)
Ability to drive	2 (4.9)	0 (0)
Accomplish goals	2 (4.9)	0 (0)
Detached from reality	2 (4.9)	0 (0)
Diet	1 (2.4)	0 (0)
Financial	1 (2.4)	0 (0)
Flat affect	1 (2.4)	0 (0)
No impact	0 (0)	9 (22.0)

[a]Patients may have reported more than 1 impact.

[b]To assess impacts experienced during lifetime, all instances of an impact were tallied across impacts experienced at diagnosis, impacts experienced during the past 30 days, changes in impacts while taking the study drug, and other impacts elicited during the interview.

Table S5. **Coping Mechanisms**

Coping Mechanisms, n (%)	Patients, N=41
**Positive**	**36 (87.8)**
Entertainment	22 (53.7)
Social activities/social support	14 (34.1)
Physical activities	13 (31.7)
Mindfulness/meditation	12 (29.3)
Religion	7 (17.1)
Therapy	6 (14.6)
Pets	5 (12.2)
Housework	4 (9.8)
Self-isolation	3 (7.3)
Marijuana use	2 (4.9)
Smokes cigarettes	2 (4.9)
Employment	1 (2.4)
Sleeps until symptoms pass	1 (2.4)
**Negative**	**24 (58.5)**
Smokes cigarettes	16 (39.0)
Alcohol/ marijuana use	5 (12.2)
Self-isolation	4 (9.8)
Sleeping too much	2 (4.9)
Repress memories	2 (4.9)
Fight others	1 (2.4)
Masking symptoms/impacts	1 (2.4)
Kissing self	1 (2.4)
Talks back to voices	1 (2.4)
**None**	**3 (7.3)**

Table S6. Drawbacks of Previous Medications

Drawbacks, n (%)	N=41
**Physical health[a]**	**39 (95.1)**
Lack of energy/drowsy	23 (56.1)
Weight gain	19 (46.3)
Involuntary movements	8 (19.5)
Trouble sleeping	7 (17.1)
Queasy stomach	7 (17.1)
Dizziness/disoriented	7 (17.1)
Dry mouth	6 (14.6)
Restlessness	4 (9.8)
Stiffness/tightness	4 (9.8)
Headaches	3 (7.3)
Gassy	2 (4.9)
Pounding heart	2 (4.9)
Numbness	2 (4.9)
Milk production	2 (4.9)
Blurry vision	2 (4.9)
Drooling	2 (4.9)
**Changes in emotions/behaviors**	**17 (41.5)**
Change in mood	8 (19.5)
Increased appetite/compulsive eating	7 (17.1)
Lack of emotions	2 (4.9)
Antisocial	2 (4.9)
Exacerbated schizophrenia symptoms	2 (4.9)
Loss of appetite	2 (4.9)
Talking to self	1 (2.4)
**Cognitive symptoms**	**5 (12.2)**
Not thinking clearly	2 (4.9)
Slurred speech/stutter	2 (4.9)
Forgetful	1 (2.4)

[a]Reported negative effects on physical health by one patient each (n=1; 2.4%): Lock jaw, low white blood cell count, erectile dysfunction, swallowing, trouble breathing, itchiness, nosebleeds, weakness, and pain.

Table S7. Treatment Aspects Most in Need of Change

Symptom/Impact, n (%)	Primary Aspect N=41	Secondary Aspect N=23[c]
Hallucinations		
Auditory	13 (31.7)[a,b]	6 (26.1)
Both (equally)	4 (9.8)	0
Visual	3 (7.3)	1 (4.3)
Social/relationships	5 (12.2)	2 (8.7)
Ability to function	3 (7.3)	1 (4.3)
Anxiety	2 (4.9)	1 (4.3)
Delusions	2 (4.9)[a]	—
Paranoia	2 (4.9)	3 (13.0)
Physical functioning	2 (4.9)[b]	2 (8.7)
Depression	1 (2.4)	
Mood	1 (2.4)	2 (8.7%)
Motivation	1 (2.4)	—
Negative thoughts	1 (2.4)	—
Stability in thinking	1 (2.4)	—
Concentration	—	1 (4.3)
Family	—	1 (4.3)
Psychotic episode	—	1 (4.3)
Self esteem	—	1 (4.3)
Talking to self	—	1 (4.3)
No aspect to improve	2 (4.9)	—

Middle column does not add to 41 as patients could provide more than one response:
[a]One patient (2.4%) reported delusions and auditory hallucinations as equal.
[b]One patient (2.4%) reported physical functioning and auditory hallucinations as equal.
[c]Sixteen patients (39.0%) did not provide a secondary aspect of importance.
Three patients (7.3%) provided a third aspect of importance: “energy level,” “a relationship with God,” and “depression.”

Table S8. Symptoms Alleviated with Study Drug

Symptom, n (%)	Participants (N=41)
Auditory hallucinations/hearing voices	26 (63.4)
Concentration	14 (34.1)
Paranoia	13 (31.7)
Anxiety/worry	9 (22.0)
Depression	8 (19.5)
Visual hallucinations	7 (17.1)
Delusions	4 (9.8)
Mood swings	3 (7.3)
Racing thoughts	3 (7.3)
Suicidal thoughts	3 (7.3)
Headaches	2 (4.9)
Violent behavior	2 (4.9)
Irritability/agitation	1 (2.4)
Muscle tension	1 (2.4)
Negative self-talk	1 (2.4)
Personality	1 (2.4)

Table S9. Change in Impact with Study Drug Treatment

Change in Impact, n (%)	N=41
Emotional or mental well-being	26 (63.4)
Self-esteem	25 (61.0)
Social activity	25 (61.0)
Relationships	21 (51.2)
Daily activities	19 (46.3)
Sleep	17 (41.5)
Hospitalizations	15 (36.6)
Physical health	15 (36.6)
Weight	11 (26.8)
General quality of life	9 (22.0)
Work	7 (17.1)
Energy	5 (12.2)
School	4 (9.8)
Stability	4 (9.8)
Coping	2 (4.9)
Incarceration	1 (2.4)
